# The molecular study of IFNβ pleiotropic roles in MS treatment

**Published:** 2013

**Authors:** Maryam Kay, Zohreh Hojati, Fariba Dehghanian

**Affiliations:** 1 MSc Student, Department of Biology, School of Sciences, University of Isfahan, Isfahan, Iran; 2 Assistant Professor, Department of Biology, School of Sciences, University of Isfahan, Isfahan, Iran

**Keywords:** Interferon Beta, MS Treatment, Cytokine Shift, Blood Brain Barrier, MHC II

## Abstract

Multiple sclerosis (MS) is one of the most important autoimmune diseases recognized by demyelination and axonal lesion. It is the most common cause of disability in the young population. Various immunomodulatory and immunosuppressive therapies, including different formulations of interferon beta (IFNβ), glatiramer acetate (GA), mitoxantrone, and natalizumab are available for this disease. However, interferon has been the best prescribed. Although the precise mechanism of IFNβ is unclear, many studies indicate some potential mechanism including blocking T cells activation, controlling pro- and anti-inflammatory cytokine secretion, preventing activated immune cell migration through BBB, and inducing repair activity of damaged nerve cells by differentiating neural stem cells into oligodendrocytes. These molecular mechanisms have significant roles in IFNβ therapy. More researches are required in order for us to comprehend the mechanism of action of IFNβ, and improve and develop drugs for more efficient MS treatment.

## Introduction

### Treatment for Sclerosis

Multiple sclerosis (MS) is the most common chronic disease of the central nervous system (CNS) which occurs in the young adult population. Globally, the median estimated prevalence of MS is 30 per 100000 and the total estimated number of people diagnosed with MS is approximately 1.3 million.^1^ Both genetic and environmental factors are thought to play roles in MS pathogenesis.^2–5^ MS is an immune-mediated process, in which infiltration of immune cells across the blood brain barrier (BBB), invasion into the brain parenchyma, demyelination, and oligodendrocytes’ death are the most important causes of the lesions. There are four clinical courses of MS including relapsing remitting (RRMS), secondary progressive (SPMS), relapsing progressive (RRMS), and primary progressive (PPMS). RRMS is the most common form of MS and affects up to 85% of the infected population.^2^ There is no cure for MS, but immunomodulatory therapies, including different formulations of interferon beta (IFNβ), glatiramer acetate (GA), mitoxantrone and natalizumab (anti-very late-4 (VLA-4 antibody) are available, of which interferon has been the best prescribed (Table 1).^2, 5, 6^

**Table 1:** FDA approved drugs for MS

Interferon β^1^
IFNβ 1b (Betaseron)
IFNβ 1b (Ziferon)
IFNβ 1a (Avonex)
IFNβ 1a (Rebif)
IFNβ 1a (Cinnovex)
Glatiramer acetate^2^
Natalizumab^3^

1 FDA approved for treating RRMS

2 FDA approved for treating RRMS SPMS

3 Anti-VLA4 antibody; temporarily suspended from the market pending safety analysis

### The pathological consequences of MS

Today, scientists believe that the first trigger factor in MS is the activation of immune cells directly against CNS antigens. However, the identity of antigens is unclear yet. There are some evidence that support the role of myelin base protein (MBP), proteolipid protein (PLP), and myelin oligodendrocytes glycoprotein (MOG) in inducing immune responses.^2^ Viral infection, including human herpes virus 6, Epstein-Barr virus, varicella-zoster virus, and herpes simplex virus are thought to be second trigger factors. However, no single virus has been clearly identified as a trigger factor. Studies show that a virus can trigger immune response by the mechanism of viral proteins mimicking the host's endogenous proteins and causing CNS inflammation.^2, 7^

There are some important pathological consequences in inflammatory lesions. Initially, direct block in nerve signals has been seen to occur by pro-inflammatory cytokines. This temporary blocking can be eliminated after immunomodulatory cytokine production. The second and the most recognized pathological consequence is demyelination that reduces the efficiency of signal conduction and threatens the health of the axon by destabilizing the structure of the axonal cytoskeleton.^3^ Axonal recovery arises after demyelination and consists of a two-step process. The first step is the axonal adaptation, which involves redistribution of sodium channels that cause sustained but slow transmission across the demyelinated segments.^3^ The second stage is remyelination mediated by surviving oligodendrocytes and depends on different factors, including switching the balance of the pro-inflammatory and anti-inflammatory cytokines by immunomodulatory process and growth factors such as IL-4 and IL-10.^3, 4^ Remyelinating oligodendrocytes show shortened internodes and thin myelin, which cause stable conduction at an almost normal speed. The loss and subsequent restoration of myelin, and neural action have been seen in early MS, but axonal transaction, as final pathological consequence, has been seen in progressive MS.^3^

### Interferon beta (IFNβ)

IFNs were discovered in 1957.^4^ The term interferon originally described the biological activity of soluble substance which interfered with viral replication.^2^ IFNs are proteins produced by cells in response to antigenic stimulation like viral RNA, bacterial product or tumor proteins. There are three classes of interferon based on the origin of the cells. Leukocytes exposed to viruses secreted IFNα, fibroblast secreted IFNβ, and lymphocytes mainly secreted IFNγ.^2^ IFNβ has pleotropic effects, including antiviral, antitumor, and anti-inflammation. After viral infection, IFNβ stimulates the expression of multiple genes, which impair the viral infection. 2’,5’ oligoadenylate synthetase (2’,5’ OAS) expression is induced in response to IFNβ, and it catalyzed the polymerization of ATP, which activates cellular ribonuclease (RNase) that cleaves viral RNA and inhibits viral protein translation.^2, 8, 9^ IFNβ also activates protein kinase R (PKR) which inhibits eukaryotic initiation factor 2 (eIF2) by phosphorylating its α-subunit which leads to decreasing of viral and, to a lesser extent, host protein synthesis. The antitumor effect of IFNβ is directly mediated by effects on proliferation, cell cycle, or apoptosis, and indirectly by immune activation.^2^ IFNβ also has anti-inflammatory effects, which make it the most important drug for MS patients.

### Interferon beta signaling pathway

**Figure 1:**
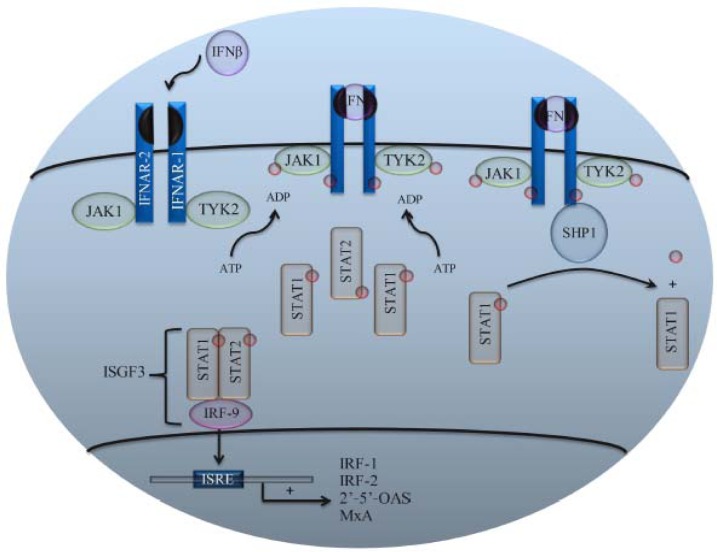
Interferon beta signaling pathway

IFNβ initiates its biological effects by binding to multisubunit receptors on the cell surface. IFNβ receptor (IFNAR) is composed of two subunits, IFNAR-1 and IFNAR-2. These receptors have inherent enzymatic activities, which lead to auto- or cross-phosphorylation of the receptor subunits.^10, 11^ Binding of IFNβ to the IFNAR causes rapid phosphorylation of the receptor's subunit, and activation of Janus Kinases (JAK1) and Tyrosine Kinases (TYK2) through auto-phosphorylation. IFNAR-1 is associated with TYK2, whereas IFNAR-2 is associated with JAK1. Phosphorylation of the IFNAR subunits converts them to the docking site for other regulatory proteins like signal transducers and activators of transcription (STAT) proteins.^12, 13^ The JAK1/TYK2 associated with IFNAR can phosphorylate STAT 1, 2, and 3. A complex is formed by Phosphorylated STAT2 and STAT1, which is associated with the DNA binding protein called interferon regulatory factor 9 (IRF9). The STAT 1:2: IRF9 complex is a transcription factor (IFN-stimulated gene factor, ISGF3), which translocates to the nucleus and binds to the IFN-stimulated response element (ISRE) of multiple genes. Different kinds of genes are targeted by ISGF3 complex, including IRF-1 (a positive regulator of IFN genes), IRF-2 (a negative regulator of IFN genes), and IFNβ, 2’, 5’ OAS, and MxA that have antiviral, antitumor, and anti-inflammation effects.^11–13^ Each signaling pathway has a negative control system. One way of inhibiting JAK/STAT signaling pathway is by tyrosine kinase dephosphorylation. SHP1 is a tyrosine phosphatase which is associated with the IFNAR-1 subunit and dephosphorylates JAK1, thus inhibiting signal transduction (Figure 1).^2, 13^

### Molecular defects in IFNβ signaling and MS

There is much evidence that confirm the decreasing of IFNβ production in MS patients.^14^ As a result, the level of IFNB-stimulated genes, especially IFNβ (IFNβ stimulate their own secretion) production, are eliminated.^15^ As mentioned before, after IFNβ binds to its receptor there is a rapid formation of ISGF3 complex, and elevation of several gene transcriptions, such as IRF1, IFNβ bind to their receptors and trigger JAK-STAT signaling pathway which promote antiviral, antitumor, and anti-inflammation effects (Details are given in the text).

IRF2, and MxA, has been seen.^11^ According to the low level of IFNβ, protein products of these genes are low in MS patients in comparison to healthy individuals.^15^ A significantly reduced level of IRF1 mRNA is reported in MS patients. In addition, the IRF1/IRF2 mRNA ratio is also lower in MS patients. Increased mRNA production of IRF2 as an inhibitor of several IFNβ-induced gene products imply that it may have an important role in decreasing IFNβ signaling pathway.^15^ There are additional abnormalities in tyrosine phosphorylation of STAT1.^2^ During normal conditions, SHP1 phosphatases remove P-Tyr-STAT1 allowing recycling of substrate for further IFNβ signaling. However, in MS patients the level of P-Tyr-STAT is increased. This can be caused by reduced level of SHP1.^13, 15^ IFNβ treatment partially reverse the IFNβ-signaling defects and increase level of IRF1, 2’,5’ OAS, and other related genes.^2^

Although the precise mechanism of IFNβ actions remains unclear, there are several proposed mechanisms of action, which this review tries to explain. As will be explained later, IFNβ suppresses inflammatory responses in MS patients by four major mechanisms, including blocking T cell activation, controlling pro- and anti-inflammatory cytokine secretion, preventing activated immune cell migration through BBB, and inducing repair activity of damaged nerve cells by differentiating neural stem cells into oligodendrocytes.^3, 5, 16^

### IFNβ Block T cell activation

T cell activation occurs via two steps. First, cell-surface receptors recognize antigen in association with MHC II molecules. Then, T cells are completely activated by several co-activator molecules, like CD40/CD40L and B7/CD26.^3, 5^ After their activation T cells differentiate into T helper and T regulatory cells. T helper cells can be subdivided into two main groups, including Th1 and Th2. These regulate the type of immune response and can cross-regulate each other. Th1 cells are pro-inflammatory which activate immune cell response by producing pro-inflammatory cytokines, like IL2 and IFNγ, whereas Th2 cells promote humoral immunity.^17^ Overactive Th1 cells can promote inflammatory types of immune disease like MS. It could be concluded that T cell inactivation might be proposed as a potential treatment for MS. Th1 and Th2 are antagonistic; each population produces cytokines that stimulate differentiation of naive T cells of the same population and suppress other population differentiations. T regulatory cells inhibit the pathogenic effect of Th1 cells by secreting anti-inflammatory cytokines.^5^ IFNβ therapy inhibits T cell activation in several ways. First, by downregulating the expression of MHC II and inhibiting co-stimulating factor (CD40/CD40L, b7/CD28) interaction IFNβ decreases T cell activation.^3, 18^ Finally, IFNβ promotes CTLA4 and death receptor (e.g. Fas) expression and induces activated T cell apoptosis.^19^

### Downregulation of MHC II expression

As mentioned earlier, the presentation of antigen to T cells occurs by MHC II molecules and this is recognized as a primary signal to activate T cells. MHC II molecules expressed on cells that serve as APCs such as macrophages, monocytes, dendritic cells and B cells.^20^ IFNβ affects the positive regulatory pathway of MHC II expression, leads to a decrease in MHC II expression, and inhibits T cell activation. MHC II expression is regulated by inducer factors, like IFNγ (positive regulatory pathway), and inhibitor factors, like IFNβ (negative regulatory pathway).^21, 22^

**Figure 2:**
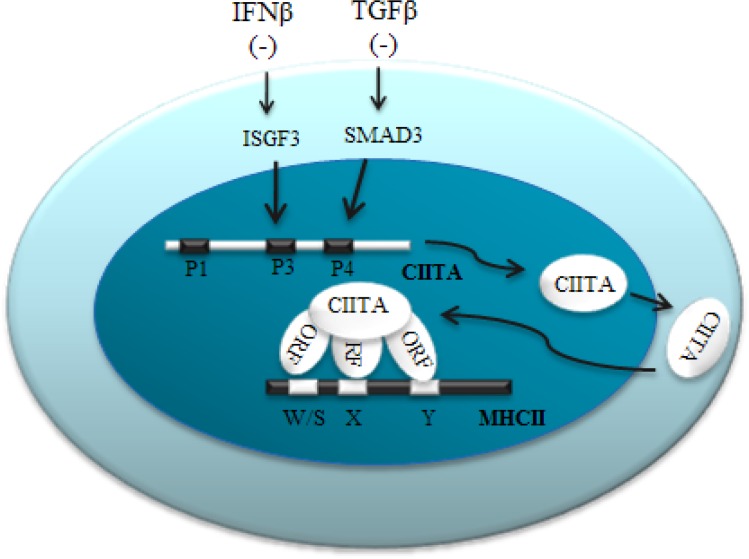
Molecular regulation of MHC class II

The promoter of MHC II contains three conserve elements [S (also W or Z), X, and Y] which are necessary for its gene expression. Binding proteins to these elements must occur in a spatially-restricted fashion to allow direct interaction among them, and for co-activators to form the active transcriptosome complex.^20^ Among these factors CIITA plays an important role in regulation of MHC II expression. Positive or negative regulatory processes typically target CIITA expression which then target MHC II expression. The expression of CIITA under inflammatory conditions parallels the expression of MHC II molecules. CIITA transcription is upregulated by IFNγ and IL4, whereas it is downregulated by IFNβ, IL10, and TGFβ. CIITA has four distinct promoters which cause cell specific expression. P1, P3, and P4 are used in dendritic cells, B cells, and non-hematopoietic cells, respectively. However, P2 is not clearly defined. Negative regulators including IFNβ and TGFβ suppress P3 and P4 expression that inhibit MHC II expression on APCs and prevent T cell activation. In addition, IFNβ indirectly affects the Suppression of CIITA P4 expression by TGFβ requires SMAD3, while IFNβ requires ISGF3 for CIITA expression suppression. CIITA is a positive regulator for MHC II expression and promotes MHC II expression by other regulatory factor (ORF) cooperation Figure 2).

### Inhibition of co-activator interaction

**Figure 3:**
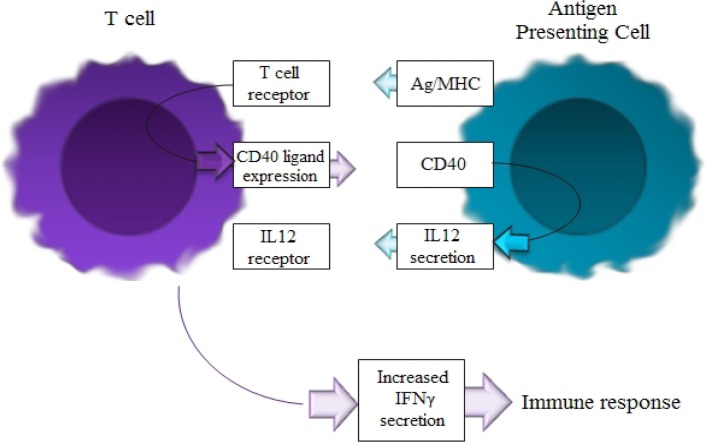
The CD40:CD40 ligand response

After the initial step of T cell activation by MHC II/TCR interaction, co-activator interactions complete T cell activation. The role of accessory molecules of CD40 and CD40L has been explored in MS. In normal conditions, antigen specific recognition events lead to activation of both T cell and APC which in turn elevate CD40 expression on the APC and CD40L on T cells. These events also induce IL12 expression by APC and IL12R expression on T cells which induces IFNγ and B7 secretion, thus promoting immune cell activation. In MS, the pattern of activation remains intact, but there is an enhancement in response to each step. Downstream events, including IL12, IL12R, and IFNγ expressions, which are mediated by the CD40/CD40L interaction, are downregulated by IFNβ. As a result, IFNβ can modulate immune response (Figure 3).^3, 17^

### Upregulation of Death receptors and CTLA4 on T cell surface

Studies indicate that death receptors (like Fas and CTLA4 receptors) are upregulated by IFNβ which induces apoptosis in activated T cells.

Fas receptor (also called Apo1 or CD95) is a death domain-containing member of TNFR (tumor necrosis factor receptor) superfamily.^23^ The Fas receptor trimerizes upon binding to FasL (Fas Ligand) and induces apoptosis through a cytoplasmic domain called DD (Death Domain). These DD domains interact with signaling adaptors like FADD (Fas-associated death domain) which carries a DED (death effector domain). This interaction can recruit the inactive DED containing procaspase-8 protein. Then, procaspase-8 is proteolytically cleaved and produces activated caspase 8, and also helps the activation of caspase 10. Activated caspase 8 and caspase 10 trigger the activation of downstream effector caspases, including caspase 3, 6, and 7. Activated procaspase-8 triggers cellular apoptosis through two separate pathways. First, procaspase-8 cleaves BID (Bcl-2 interacting protein), and its C-terminal part is translocated to mitochondria and triggers mitochondrial apoptosis pathway. Another pathway is that procaspase-8 cleaves procaspase-3 directly and induces apoptosis signaling pathway.^24, 25^

**Figure 4:**
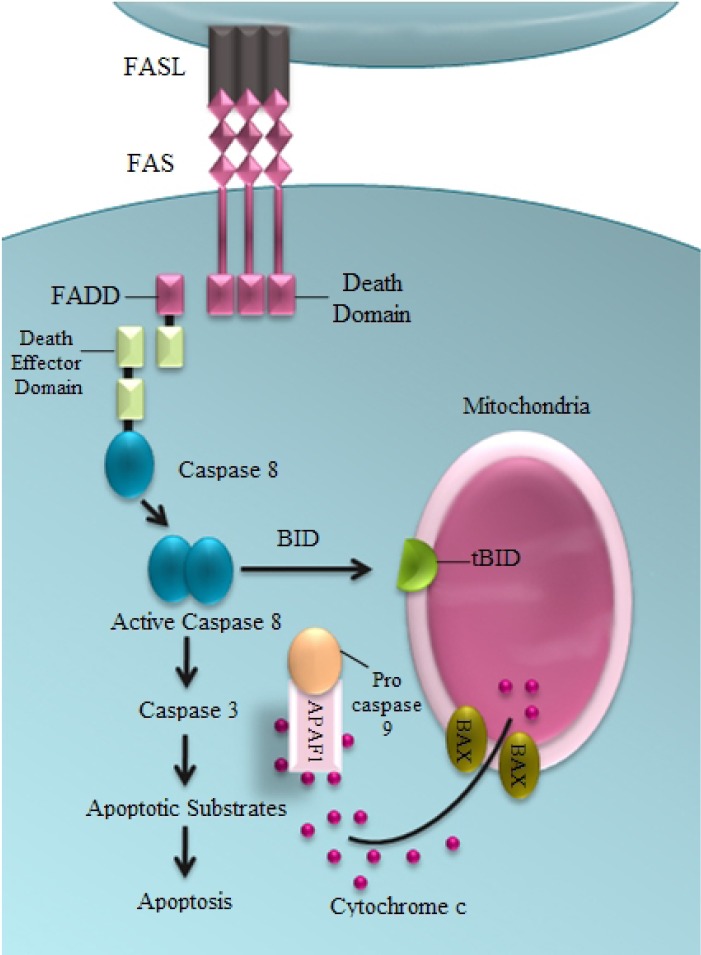
Two Fas-mediated apoptotic pathways

CTLA-4 preserves PI3K activity and inhibits Akt by activation of the phosphatase PP2A. This process causes cell cycle arrest, apoptosis, and lack of energy in the target cell (Figure 4).

**Figure 5:**
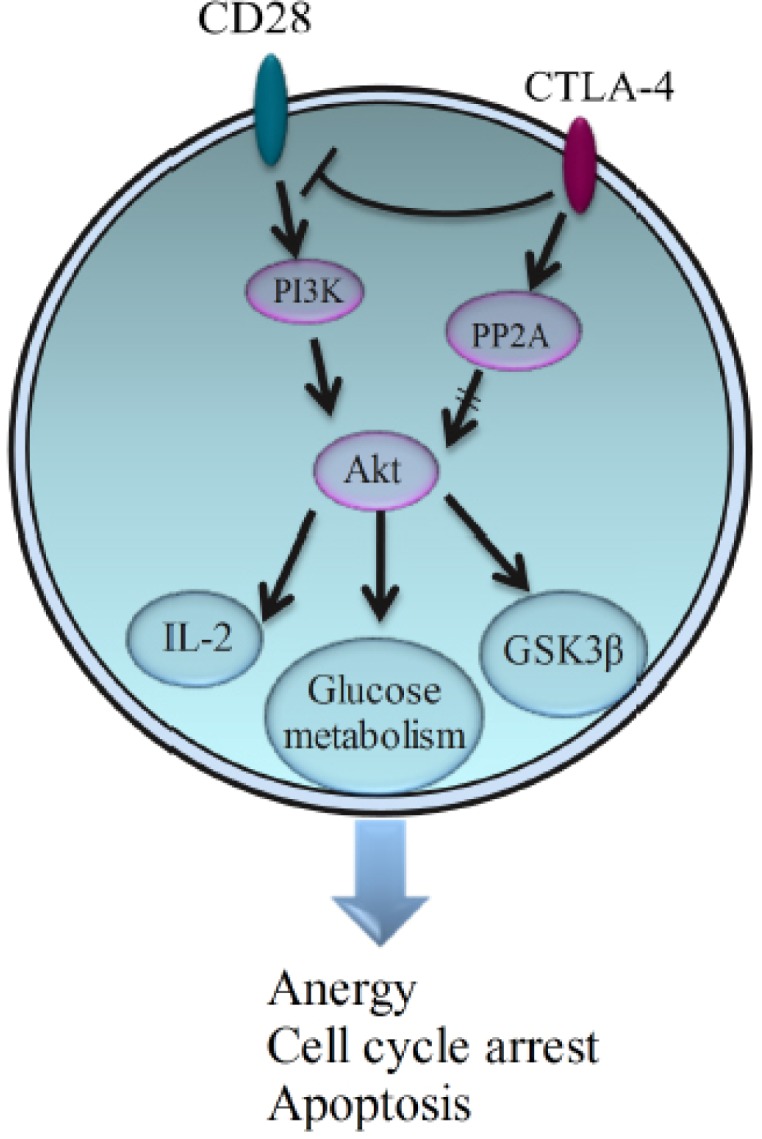
CTLA-4 mediated T cell inhibition pathway

The B7 families of co-stimulating molecules have important roles in the regulation of T cell activation. In addition to CD28, CTLA4 is also a member of B7 family, which is T cell- restricted and only appears after T cell activation. T cell activation is promoted by CD28 induced PI3K/Akt signaling pathway, which leads to cell activation, proliferation, and survival. After T cell activation, CD28 downregulates CD28 production and promotes CTLA4 expression. Akt plays an important role in cellular metabolism and activation, and is directly targeted by CTLA4. CTLA4 activates a type II serine/threonine phosphatas PP2A, which can reverse PI3K-mediated phosphorylation of Akt (Figure 5). Akt inactivation by PP2A cause downstream pathway inhibition that lead to anergy, cell cycle arrest, and apoptosis.^26–28^

### IFNβ alters cytokine shift

Autoreactive Th1 cells are the primary drivers of the pathogenesis of MS that are activated by APCs. Subsequently, these cells release pro-inflammatory cytokines, which promote the upregulation of adhesion molecules and their ligands on the BBB endothelium cells to enhance activated immune cell migration. As immune cells inter the CNS, activated T cells secrete more pro-inflammatory cytokines, which activate CNS inflammatory cells (like monocytes and macrophages). This leads to demyelination and axonal lesions through Fc-madiated phagocytosis and neurotoxic substances (e.g. free radicals, nitric oxide, and glutamate).^5, 29^

These pro-inflammatory processes may be controlled by Th2-mediated anti-inflammatory cytokines (like IL4 and IL10) that lead to deactivation and initiation of recovery phases.^30^ IFNβ can change the shift of pro- and anti-inflammatory cytokines through increasing anti-inflammatory and deceasing pro-inflammatory cytokines. The most important cytokines involved in development and activation of Th1 cells are IL2 and IL12. IL2 is known as T cell growth factor, and T cells are the main target of this cytokine. IL2 also has an important role in monocytes, macrophage, and B cell activation. IL2 induces IFNγ expression by T cells that leads to MHC II overexpression on the surfaces of the APC cells. IL2 and IL2R expression is highly upregulated in MS patients.^2^ IL12 expressed by APCs cause IFNγ production in T cells and macrophages that are essential for complete immune response. Other roles of IL12 are T cell growth, co-stimulatory and anti-apoptotic activities, suppression of the production of cross-regulatory cytokines such as IL-4 and IL-10.^31^

TNFα and TNFβ play important roles in tissue damage in MS. They are produced by activated monocytes, macrophage, and B and T lymphocytes, and are toxic in CNS.^2^ IFNβ inhibits the production of IL2, IL12, and TNF, and reduces IL2R expression on the surface of the cells that inhibit immune cell activation and sustain CNS cells from damage.^5, 2^ On the other hand, IFNβ increase expression of anti-inflammatory cytokines like IL4, IL10, and TGFβ. As was previously mentioned, Th1 and Th2 play important roles during immune responses. In the initial phase of inflammation Th1 responses are dominant, whereas Th2-mediated responses become important in the recovery phase. IFNβ therapies inhibit Th1 responses by decreasing pro-inflammatory cytokines, and promote Th2 responses by increasing anti-inflammatory cytokines. Another cell type that limits inflammatory response is the CD8^+^/CD28^+^ T suppressor cells that are mediated by several cytokines, such as IL4, IL10, and TGFβ. Ts activity is decreased in MS patients promoted by IFNβ activities.^2^

### The effect of interferon beta in blood-brain barrier

As stated previously, MS lesions emerge as activated immune cells, pas through BBB, and recruit more immune cells in CNS that cause inflammation, demyelination, and neuronal damages. Different cytokines, adhesion molecules, integrins, and MMPs have important roles in BBB permeability and lesion formation.^5^ IFNβ control the expression of these factors by several mechanisms. IFNβ prevents T cell adhesion to endothelium cells and as a result inhibits immune cell migration to CNS. IFNβ mediate cleavages of VCAM expressed on the BBB endothelial cells and convert them into sVCAM (soluble vascular cell adhesion molecule) that prevent adhesion of leukocyte to endothelial cells. SVCAM molecules can bind competitively to VLA-4 receptors on the surface of T cells and obstruct binding of T cells to endothelium. This process can only prevent T cell maturation and migration, whereas VCAM/VLA4 complex can promote hematopoiesis and peripheral T cell apoptosis. IFNβ also downregulate VLA4 expression on the surface of CD4^+^/CD8^+^ T cells to reduce immune cell traffic along BBB and thus improve brain lesion of MS patients.^32^

CD73 is an ectoenzyme expressed on endothelial, epithelial, and peripheral blood lymphocytes. CD73 is responsible for the production of adenosine. During inflammation, ATP is released from damaged cells and converted to AMP by CD39 in extracellular environment. This AMP is converted into adenosine by CD79.^33^ Studies show anti-inflammatory and immunosuppressive functions for adenosine. Extracellular ATP is a pro-inflammatory mediator inducing immune cells to produce inflammatory cytokines, such as IL-12, IL-18, and TNFα. Adenosine inhibits inflammatory cytokine production and promotes induction of IL-10. Thus, adenosine acts as a negative feedback signal to prevent the immunostimulatory effect of ATP. Some studies have approved that CD73 can also improve endothelial barrier function. Therefore, IFNβ can control leukocyte migration and inflammatory cytokine production by increasing CD73 expression.^33, 34^

**Figure 6:**
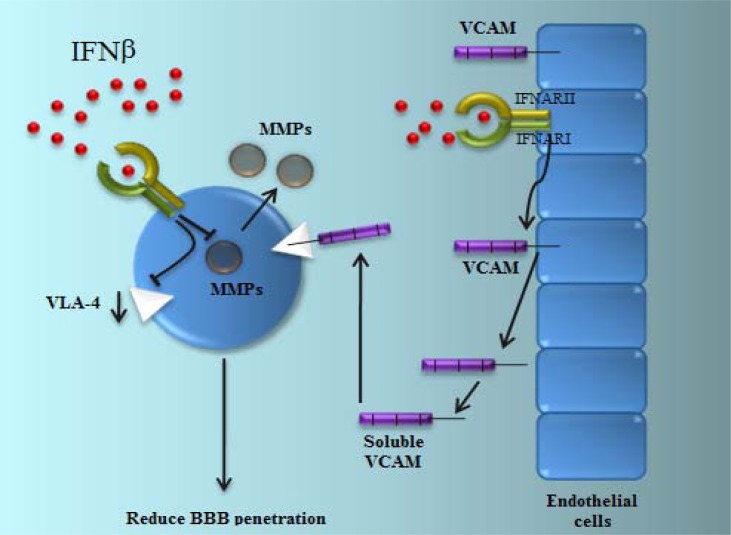
IFNβ diminishes the ability of activated T cells to cross the blood brain barrier and enter the central nervous system parenchyma

MMPs are endopeptidases that have significant roles in BBB permeability. These endopeptidases are secreted by activated T cells and macrophages and facilitate their migration to CNS by disruption of the BBB. In MS patients, levels of serum MMP-9 are increased, and the concentration of tissue inhibitors of MMP-type-1 (TIMP-1), which are anti-inflammatory glycoproteins, is decreased (Figure 6). IFNβ therapy increases TIMP-1, whereas it decreases MMP-9 expression. Studies indicate that MMP-9 facilitate lesion formation while TIMP-1 induce remyelination and neural repair.^5^ IFNβ also upregulate N-cadherin and vinculin expression, which are junctional proteins, and decrease BBB permeability. Expression of HLA molecules on the surface of endothelia cells is another significant feature of MS. This process converts HLA molecules into antigen presenting cells, which induce inflammation in CNS. INFβ downregulates MHC II expression in order to control immune responses. As a result, INFβ can inhibit immune cell migration and suppress inflammation in CNS.^35^

In conclusion, IFNβ controls immune cell migration by the downregulation of VLA-4, MHC II, and MMPs expression. Moreover, it upregulates TIMP-1, CD73, N-cadherin, and vinculin expression that decrease BBB permeability and prevent immune cell migration.

### The effect of interferon beta in the central nerve system

Studies show a local expression of INFβ in the site of injury, where it suppresses chemokine secretion by activated microglia in CNS. This results in a reduction of lymphocyte migration through BBB and also reduces uptake and presentation of antigen by macrophage and dendritic cells.^36, 37^ Activated microglia in cortical neurons secrete some inflammatory cytokines, such as TNF-α, IL-1β, glutamate, superoxide, and nitric oxide, which are all neurotoxin factors and cause neural injury in MS patient. IFNβ can suppress secretion of superoxide onions and glutamate by 20%-30% and prevent apoptosis.^5^

IFNβ both reduce production of pro-inflammatory cytokines and decrease antigen presentation by CNS resident cells. Nevertheless, IFNβ has more significant roles in proliferation, differentiation, and survival of neural stem/progenitor (NPCs) cells in CNS. Oligodendrocytes derived from hNPC differentiation, are capable of repairing damaged brain lesions via remyelination. Accordingly, the role of IFNβ in Oligodendrocyte's differentiation from hNPC has an important effect on the therapeutic strategy for improvement of demyelinating neurons.^16^ Researchers have shown that IFNβ sustain hNPC proliferation and differentiation, and protect cells from apoptosis. HNPC cells produce IFNβ receptors and IFNβ initiate proliferation or differentiation pathways depending on IFNβ concentration. In low concentration, IFNβ sustains proliferation and promotes differentiation into astrocytes, whereas in higher concentration it sustains proliferation and promotes differentiation into Oligodendrocytes. Further super array analysis reveals the main signaling pathway involved in the above functions. Studies show that STAT1 and STAT2 proteins significantly increase (more than 7-fold). This proves the main role of JAK/STAT signaling pathway in the upregulation of the expression of target genes that are involved in proliferation, survival, and differentiation of hNPCs.^16, 38^ Some of the genes that are upregulated by IFNβ are Fas (associated with NPC survival), NRG2 (involved in neuron growth), and FGF2 (associated with NPC self-renewal).^39–41^ Upregulation of these genes by IFNβ causes oligodendrocyte differentiation and helps to repair damaged neurons.^16, 38^
